# Microbiota epitope similarity either dampens or enhances the immunogenicity of disease-associated antigenic epitopes

**DOI:** 10.1371/journal.pone.0196551

**Published:** 2018-05-07

**Authors:** Sebastian Carrasco Pro, Cecilia S. Lindestam Arlehamn, Sandeep K. Dhanda, Chelsea Carpenter, Mikaela Lindvall, Ali A. Faruqi, Clark A. Santee, Harald Renz, John Sidney, Bjoern Peters, Alessandro Sette

**Affiliations:** 1 La Jolla Institute for Allergy and Immunology, Department of Vaccine Discovery, La Jolla, California, United States of America; 2 University of California San Francisco, Department of Medicine, San Francisco, California, United States of America; 3 Institute of Laboratory Medicine, Philipps Universitaet Marburg, Marburg, Germany; 4 University of California San Diego, Department of Medicine, La Jolla, California, United States of America; University of Manitoba, CANADA

## Abstract

The microbiome influences adaptive immunity and molecular mimicry influences T cell reactivity. Here, we evaluated whether the sequence similarity of various antigens to the microbiota dampens or increases immunogenicity of T cell epitopes. Sets of epitopes and control sequences derived from 38 antigenic categories (infectious pathogens, allergens, autoantigens) were retrieved from the Immune Epitope Database (IEDB). Their similarity to microbiome sequences was calculated using the BLOSUM62 matrix. We found that sequence similarity was associated with either dampened (tolerogenic; e.g. most allergens) or increased (inflammatory; e.g. Dengue and West Nile viruses) likelihood of a peptide being immunogenic as a function of epitope source category. Ten-fold cross-validation and validation using sets of manually curated epitopes and non-epitopes derived from allergens were used to confirm these initial observations. Furthermore, the genus from which the microbiome homologous sequences were derived influenced whether a tolerogenic versus inflammatory modulatory effect was observed, with *Fusobacterium* most associated with inflammatory influences and *Bacteroides* most associated with tolerogenic influences. We validated these effects using PBMCs stimulated with various sets of microbiome peptides. “Tolerogenic” microbiome peptides elicited IL-10 production, “inflammatory” peptides elicited mixed IL-10/IFNγ production, while microbiome epitopes homologous to self were completely unreactive for both cytokines. We also tested the sequence similarity of cockroach epitopes to specific microbiome sequences derived from households of cockroach allergic individuals and non-allergic controls. Microbiomes from cockroach allergic households were less likely to contain sequences homologous to previously defined cockroach allergens. These results are compatible with the hypothesis that microbiome sequences may contribute to the tolerization of T cells for allergen epitopes, and lack of these sequences might conversely be associated with increased likelihood of T cell reactivity against the cockroach epitopes. Taken together this study suggests that microbiome sequence similarity influences immune reactivity to homologous epitopes encoded by pathogens, allergens and auto-antigens.

## Introduction

Several studies indicate that similarities between related and even phylogenetically distant protein sequences can influence host immune reactivity. This phenomenon, termed molecular mimicry, was originally described in Fujinami et al. [1] and is hypothesized to play an important role in the development of certain autoimmune diseases [2]. There has been a recent renewed interest in this concept, where similarity and cross-reactivity have been demonstrated for distant sources such as HIV, cytomegalovirus and herpes simplex virus [3, 4]. Furthermore, we have recently shown that cross-reactivity may also occur between influenza virus and the food allergen ovalbumin with functional relevance for the prevention of food allergic Th2 immune responses [5]. A related concept where peptide similarity drives immune recognition is the conservation of peptides in one pathogen across others. For example, in the case of *Mycobacterium tuberculosis* [6] certain epitopes that are highly reactive share significant similarity to nontuberculous mycobacteria, while in other cases extensive sequence similarity to several bacterial classes seems to be associated with loss of reactivity.

A large body of literature indicates that the host microbiome has a profound influence in shaping and modulating host reactivity and immune functioning, primarily at the level of innate and adaptive immunity [7–9]. In this context, it seems reasonable to assume that the sequence similarity of antigens recognized by the host immune response (whether infectious agents, allergens or autoantigens) might also play a role in modulating adaptive immunity.

Previous analyses [10] suggested that exposure to microbial peptide sequences can modulate immune responses to similar pathogen and allergen derived peptide epitopes. Specifically, those studies suggested that such microbiome-derived peptides might be perceived as self by the immune system and thereby be tolerogenic, resulting in the elimination or functional silencing of potentially reactive T cell clones.

Here we expanded this analysis to a broader set of epitope sources, to consider 11 different allergens, 18 viral, 4 bacterial, 1 eukaryote, and 4 autoimmune and inflammatory antigens. We found that while microbial exposure might be, in certain cases, involved in blunting potentially harmful antigen/allergen-specific immune responses, in other cases microbial sequence similarity actually increased the likelihood of immunogenicity for pathogen-derived sequences.

## Materials and methods

### Automatic assembly of a set of validated HLA class II epitopes from the Immune Epitope Database (IEDB)

Sets of HLA class II restricted epitopes were created using a method described in Carrasco Pro et al [11]. Here, we developed 38 epitope sets derived from 11 allergens, 4 autoimmune diseases and 23 infectious diseases found in the IEDB (www.iedb.org). The IEDB harvests and curates epitope data from published literature. The database does not have any consistent information related to the vaccination status of subjects. To ensure consistency in our study, all peptide sequences analyzed were 15 residues long, and any epitope longer than 15 residues was represented by all possible 15-mer components, which were then utilized to calculate their similarity to the 15-mers derived from human microbiota sequences (S1 Table).

### Generation of control sets of negative peptides

For each category (allergy, autoimmunity and infectious disease), negative peptides were derived using the sequences of the antigens found in the category’s epitope sets. The 15-mers derived from an antigen were compared to epitope sequences from the same antigen, and in order to be considered a negative peptide it was required that the 15-mer did not share more than 9 continuous residues with a given 15-mer epitope sequence. Further, an additional set of 15-mer epitopes found in the IEDB [12] that were not included in the targeted categories was created and also used for comparison to the negative peptides to ensure that each of the negative peptides was not fortuitously homologous to a positive epitope from a different organism (S2 Table).

### Manually curated dominant epitope sets

We also studied sets of dominant epitopes, derived from antigens and allergens from various target indications (S3 Table). Namely, the sets encompass panels of epitopes from several known Timothy Grass (TG) allergens, Ragweed (RAG) allergens, and antigens from house dust mites (HDM), and cockroaches (CR). In all cases, the epitopes were derived from series of studies conducted in our laboratory, with similar protocols and readouts (either *ex vivo* or *in vitro*), using ELISPOT assays measuring various cytokines (primarily IFNγ and IL-5). In all cases, the identification of these epitopes is described in peer reviewed reports [13–16]; in some cases, the epitope sets were selected on the basis of interim analyses, and do not exactly match the final epitope lists in the published reports. We also studied matching sets of “negative” control peptides (S4 Table). These negative peptides were found to completely and consistently fail to induce responses in all donors tested in the corresponding series of studies on the basis of which the positive epitopes were defined.

### Human microbiome data

Human microbiome sequences were retrieved from the Human Microbiome Project (http://www.hmpdacc.org/HMRGD/) [17, 18], where the set of annotated genomes were downloaded as protein sequences in a multi-fasta format. All possible 15-mers derived from each protein were used for the BLOSUM score calculation against the epitope sets, as described below.

### Sequence similarity scoring system

To estimate the similarity between two given sequences we used a BLOSUM matrix, which is based on the relative frequency of amino acids and their substitution probability between sets of aligned proteins, and contains log-odds scores for each of the possible substitutions of the 20 amino acids. In this study we used the BLOSUM62 matrix [19], as previously utilized for microbiome similarity studies [10], to calculate a BLOSUM score according to the formula:

$$bl(a,b)/\sqrt{bl(a,a)*bl(b,b)}$$

where a and b are the two peptides, the BLOSUM score for a and b were divided with the square root of the product of the BLOSUM score of the two peptides aligned with themselves. This formula assigns two 100% identical peptides a score of one.

### Determination of microbiome homology for epitope and negative-control sequences

For each sequence in the epitope and non-epitope sets, similarity scores of the best 15-mer for each epitope and non-epitope sequence were recorded. To ensure the consistency of our results, we performed a ten-fold cross-validation for each category. Epitopes were randomly divided into ten groups with their associated non-epitopes and a cross-validation was performed using the ten groups.

### House dust microbiota dataset

16S rRNA-based microbiota profiles derived from house dust from residences in which children developed atopy (pro-allergic) or did not develop atopy (non-allergic)[20] were used to predict the evolutionarily conserved metagenome of these microbiota as described by Okuda et al. [21], using the Kyoto Encyclopedia of Genes and Genomes (KEGG) [22, 23] and KEGG orthology (KO) identifiers. In the pro-allergic microbiota 614 bacterial taxa (16S rRNA sequences that share ≥97% DNA sequence homology) were predicted to encode 5,040 KOs. The number of taxa in the non-allergic house dust microbiota was similar, with a total of 644 taxa, the genomes of which were predicted to encode 5,149 KOs.

The predicted genomic content of both pro-allergic and non-allergic house dust microbiota largely represented overlapping KOs. Therefore, KOs were separated into three groups: 1) those unique to pro-allergic house dust microbiota (41), 2) those unique to non-allergic house dust microbiota (150), and 3) the remaining KO shared between pro-allergic and non-allergic microbiota. Next, we derived all the possible 15-mers from the protein sequences found in each of these groups of KOs, and a list of 15-mers was generated for each of the main groups. As peptides from the pro-allergic and non-allergic sets could potentially be found in both sets, we decided to compare the 15-mers per KO from each group against the other two remaining groups, to ensure the uniqueness of the sets. As an example, each 15-mer of the KOs from the non-allergic group that was found common in the shared or allergic group was discarded from the analysis.

### Statistical analyses

As the similarity scores of epitopes and non-epitopes per category were not normally distributed, a Kruskall-Wallis test was used to identify significant differences in the median similarity scores between epitopes and non-epitopes. Standard Chi-square testing was used to assess significance of difference in frequency distribution of different peptides in different genera, with appropriate Bonferroni corrections. Statistical analyses were performed in R studio and Graph Pad Prism (San Diego, US) software.

### Peptides for experimental validation of the results

The hits per category were sorted according to descending BLOSUM scores. From the 14 inflammatory categories, defined as instances where epitopes were less similar than non-epitopes to the microbiome, the top ten hits for each category were selected for analysis. In the case of nine tolerogenic categories, defined as instances where epitopes were more similar than non-epitopes to the microbiome, the top ten hits from their respective epitope sets were likewise also selected, as non-epitopes will inherently produce negative results in immunogenicity assays. Hits from the Rheumatoid Arthritis tolerogenic category were not included in this analysis because too few hits were available. The set of peptides selected for experimental testing can be found in S5 Table for the epitopes derived from the “tolerogenic” categories and in S6 Table for the epitopes derived from “inflammatory” categories. Since ten high BLOSUM peptide hits were selected for each of the 23 categories, this corresponded to a total of 230 human microbiome peptides. A third set of peptides (S7 Table) was derived from naturally processed HLA class II peptides recorded in the IEDB (n = 3231). These were predicted for binding using the previously described 7-allele method [24] and peptides not predicted to bind below the threshold of at least 20^th^ percentile were excluded (979 peptides were predicted as binders). Similarity to the microbiome was estimated by BLOSUM score calculations as described above. Peptides were purchased from Mimotopes (Clayton, Victoria, Australia) and/or A and A (San Diego, CA) as crude material on a small (1 mg) scale. Individual peptides were resuspended in DMSO to a final concentration of 20 mg/ml. Peptides were pooled (10 peptides per pool) according to their category. Each pool was at a final concentration of 2mg/ml per peptide.

### Study subjects and PBMC isolation

We recruited 10 healthy adults from San Diego, USA. All participants provided written informed consent for participation in the study. This study was performed with approvals from the Institutional Review Board at La Jolla Institute for Allergy and Immunology (protocols VD-101 and VD-071). No detailed clinical history including vaccination records is available for the participants. PBMC (peripheral blood mononuclear cells) were isolated from collected blood or leukapheresis by density gradient centrifugation, according to manufacturer’s instructions. Cells were cryopreserved in liquid nitrogen suspended in FBS containing 10% (vol/vol) DMSO.

### *Ex vivo* IFNγ/IL-10 Fluorospot

After overnight resting of the PBMCs, the response to peptide pools was measured by IFNγ and IL-10 dual Fluorospot, according to manufacturer’s instructions. Plates were coated with 5μg/ml anti-human IFNγ (1-D1K, Mabtech), and 10μg/ml anti-human IL-10 (9D7, Mabtech). Cells were plated at a density of 2x10^5^ cells/well with peptide pools (2μg/ml). PHA (10μg/ml) was used as a positive control and medium containing 0.1% DMSO (corresponding to the percentage of DMSO in the pools) was used as a negative control. Plates were cultured for 24h at 37°C and developed using anti-human IFNγ (7-B6-1-FS-FITC and anti-FITC-490, both at 1:200, Mabtech) and anti-human IL-10 (biotinylated-12G8, 2μg/ml and SA-550, 1:200, Mabtech). Fluorescent spots were counted by computer-assisted image analysis using an AID iSPOT ELISPOT reader (AID-diagnostika, Germany). To be considered positive a peptide pool response had to match all of three different criteria: 1) elicit at least 20 net spot-forming cells (SFC) per 10^6^ PBMC, 2) p≤0.05 by Student’s t-test or by a Poisson distribution test, and 3) stimulation index ≥2. No cells producing both IL-10 and IFNγ were detected.

### HLA typing

To ensure that the donors represented a diverse set of HLAs four-digit HLA typing (S8 Table) was performed at the La Jolla Institute (LJI) as described previously [25]. Here, genomic DNA was isolated from PBMC using standard techniques (REPLI-g; Qiagen). Amplicons for HLA class I and class II genes were generated using PCR and locus-specific primers. Amplicons of the correct size were purified using Zymo DNA Clean-up Kit, according to the manufacturer’s instructions. Sequencing libraries were prepared using Nextera XT reagents (Illumina), according to manufacturer’s instructions. The libraries were purified using AMPure XP (Beckman Coulter) with a ratio of 0.5:1 beads to DNA (vol/vol). The libraries were pooled in equimolar amounts and loaded at 5.4pM on one MiSeq flowcell with 1% phiX spiked in (MiSeq Reagent Kit v3). Paired-end sequencing was performed with 300 cycles in each direction. HLA typing calls were made using HLATyphon (https://github.com/LJI-Bioinformatics/HLATyphon).

## Results

### Automatic assembly of a set of validated HLA class II epitopes from the IEDB

We previously developed a method to select reference epitope sets [11] from IEDB categories (infectious disease, autoimmunity, allergy) based on epitope-associated assay information, such as the response frequency of the epitope in screened donor cohorts, type of assays capturing epitope-specific responses and epitope size. Here, we used this same methodology to select sets of HLA class II restricted T cell epitopes. In all we defined 38 sets of indications/taxonomical origins for which there were at least 20 epitopes described in the literature and that were curated in the IEDB. This number was arbitrarily considered a number sufficient to allow meaningful analysis. An exception was made in the case of Rheumatoid arthritis epitopes, where 11 epitopes were available, since preliminary analysis indicated that the results were associated with statistical significance. Specifically, we considered eleven sets of allergen epitopes derived from food allergens, various plants, fungi, insects, mites and mammals. We further considered four different sets of epitopes associated with autoimmune diseases (diabetes, rheumatoid arthritis, gluten-associated disease and multiple sclerosis). In terms of infectious disease, we selected eleven different sets of RNA viral epitopes, seven sets of DNA virus epitopes, four sets of bacterial epitopes and one set of *Plasmodium* derived epitopes.

The list of epitope sets is shown in Table 1, together with the number of epitopes found in each. For each of the 38 sets, we selected groups of control non-epitopes from the IEDB, derived from the same protein sources, that have never been reported in the published literature to be immunogenic (as detailed in the methods section). Overall, each epitope set contained a median of 125 epitopes (range 11 to 2083), and negative control sets contained a median of 2333 sequences (range 12 to 12182).

**Table 1:** List of epitopes and non-epitopes per category.

		Category	Epitopes	Non Epitope	Epitope Median Scores	Non Epitope Median Scores	p-value
Allergy							
Food							
	Plants						
		Fabaceae	89	12	0.603	0.646	3E-04
Environment							
	Plants						
		Amaranthaceae	22	1123	0.605	0.640	0.02
		Betulaceae	61	537	0.595	0.610	0.07
		Cupressaceae	23	752	0.584	0.593	0.19
		Timothy Grass	547	12182	0.621	0.615	0.85
		Other Grass	127	2182	0.782	0.604	0.72
	Fungi						
		Aspergillus	113	2395	0.599	0.626	3E-07
		Other Fungi Allergy	89	2793	0.632	0.641	0.09
	Animals						
		Insects (V + CR)	112	2079	0.595	0.601	0.07
		Arachnid (HDM)	164	4466	0.588	0.586	0.68
		Mammals Allergy (cat, dog and others)	125	1218	0.614	0.588	2E-12*
Autoimmunity							
		Diabetes	164	369	0.588	0.583	0.94
		Rheumatoid Arthritis	11	3132	0.765	0.625	0.003*
		Gluten	117	2427	0.690	0.599	2E-16*
		Multiple Sclerosis	92	307	0.570	0.588	0.001
Infectious Disease							
Virus							
	ssRNA (-) strand virus						
		Influenza AH1N1	731	3632	0.597	0.587	1E-09*
		Influenza AH3N2	296	2047	0.599	0.589	4E-05*
		Other Influenza A	726	6001	0.592	0.589	0.008*
		Influenza B	72	184	0.579	0.589	0.31
		Paramyxoviridae	112	425	0.601	0.604	0.27
	ssrNA (+) strand virus						
		Hepatitis C	901	10909	0.592	0.586	6E-04*
		Dengue	124	12014	0.611	0.594	4E-04*
		West Nile	348	2036	0.594	0.589	0.03*
		Japanese Encephalitis	99	2624	0.589	0.591	0.49
		Yellow Fever	282	2815	0.590	0.595	0.008
		Coronavirus	108	1420	0.585	0.587	0.13
	Retrotranscribing virus						
		Hepatitis B	64	886	0.601	0.572	3E-08*
	dsDNA virus						
		Adenovirus	82	205	0.575	0.588	9E-04
		Alphaherpesviridae	92	7172	0.591	0.595	0.42
		Betaherpesviridae	264	8799	0.585	0.592	0.007
		Gammaherpesviridae	177	4321	0.588	0.590	0.25
		Poxviridae	139	6549	0.594	0.595	0.37
		Papillomavirus	137	2271	0.586	0.580	0.02*
Bacteria							
	Actinobacteria/ proteobacteria						
		Alphaproteobacteria	30	4021	0.689	0.643	0.02*
		Betaproteobacteria	2083	4667	0.635	0.619	2E-16*
	Fimmicutes/ other bacteria						
		Clostridiales	358	89	0.604	0.594	0.02*
		Other Bacilli	407	367	0.609	0.615	0.14
Eukaryote							
	Alveolata						
		Plasmodium	593	4807	0.605	0.613	1E-08

No asterisk in the p-values denotes epitopes having significant median similarity score lower than the non-epitopes.

* Categories with epitope similarity scores higher than the non-epitopes.

### HLA class II restricted epitopes and control non-epitope sets vary in similarity to microbiome sequences

To investigate the potential impact of microbiome sequence similarity on epitope reactivity, we next estimated the similarity of each epitope and non-epitope to published microbiome sequences by calculating the maximum BLOSUM score for each of the sets, following the methodology described by Bresciani et al [10].

**Fig 1:**
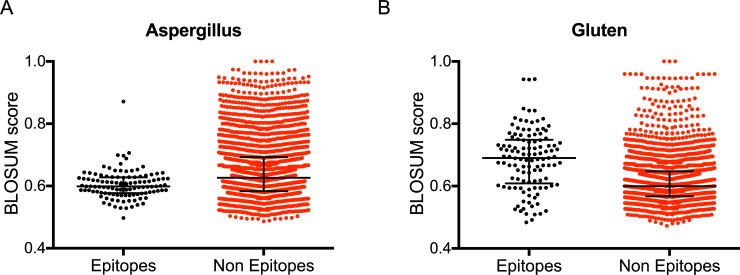
HLA class II restricted T cell epitopes vary in similarity to human microbiome sequences. Epitope (black) and non-epitope (red) maximum BLOSUM score per individual peptide are shown for the *Aspergillus* (A) and Gluten (B) epitope categories. Median ± interquartile range are shown for each distribution.

Accordingly, we calculated for each epitope in each set the maximum degree of BLOSUM similarity to any sequence contained in the human microbiome sequence set. We then calculated the p-values associated with the epitope set being less similar to the human microbiome than the matching non-epitope. In the context of the present study, this could indicate a tolerogenic influence of the microbiome. An example distribution of the epitope and non-epitope BLOSUM scores can be found in Fig 1A, where the category *Aspergillus* is represented; the median score is 0.599 for the epitopes, and 0.626 for non-epitopes (p-value 3E-07).

Conversely, we calculated the p-value associated with the epitope sets being significantly *more similar* to microbiome sequences (Table 1). This eventuality would be associated with an inflammatory or mimicry influence of the microbiome sequences on epitope immunogenicity. An example of this type of category is shown in Fig 1B, where the analysis of gluten-derived epitopes is shown.

**Fig 2:**
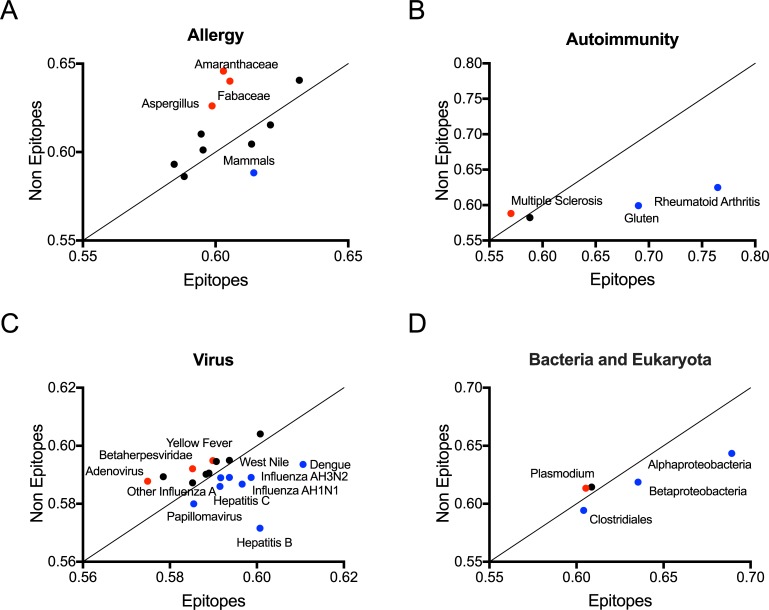
Epitopes vary in similarity to microbiome sequences. Epitope and non-epitope median BLOSUM scores for (A) Allergy, (B) Autoimmunity, (C) Virus, and (D) Bacteria categories. Blue dots indicate non-epitopes being less conserved in the human microbiome, red dots when epitopes are less conserved and black dots indicate categories with no change in conservation when comparing non-epitopes to epitopes. Diagonal line (black) indicate no change in conservation when comparing non-epitopes to epitopes.

Strikingly, significant p-values were observed for 22 out of 38 epitope sets. In eight cases, the epitope sets were significantly less similar than non-epitopes to microbiome sequences (p-values in the 1E-08 to 0.02 range). In the remaining fourteen instances, epitopes were significantly more similar than non-epitopes to microbiome sequences (p-values in the 2E-16 to 0.02 range). Fig 2 shows the ratio of BLOSUM scores between the different sets, with the categories where epitopes<non-epitopes highlighted in red and epitopes>non-epitopes highlighted in blue.

In conclusion, while significant differences between epitope and non-epitopes sets are frequently detected, the differences can be either reflective of a positive or negative influence. In general, allergen epitopes tended to be less similar, with the important exception of allergens of mammalian origin. In the autoimmune sets, diabetes and multiple sclerosis epitopes were less similar, while rheumatoid arthritis and gluten were more similar. In these cases, the microbiome sequences might have an overall “tolerogenic” or “blunting” effect on T cell responses.

RNA viral epitopes, with the exception of Yellow Fever, were more similar to microbiome sequences. In the cases where epitopes are more similar to microbiome sequences the microbiome might exert an “inflammatory” or “priming” influence. Contrasting patterns were seen in the case of DNA viruses, with epitopes from papilloma and Hepatitis B virus epitopes more similar to the microbiome, while epitopes from Adenoviruses and Betaherpes viruses were less similar. Contrasting patterns were also seen in the remaining sets, with epitopes more similar in the case of *Clostridiales*, *Alpha-* and *Beta-Proteobacteria*, and less similar for *Plasmodium*.

### Ten-fold cross-validation of the similarity differences and further validation with manually curated epitope sets and matched negative controls

To further support our results, we performed ten-fold cross-validation experiments. In these analyses, the epitope sets were partitioned in different subsets as described in the methods section. If the significant difference observed was due to sampling biases, we expected that significance would be lost in the partitioned sets. The results in Table 2 show the minimum, maximum and median p-values observed, and demonstrate that in all epitope sets for which significant differences were noted, median p-values remain significant even after tenfold validation.

**Table 2:** Ten-fold cross validation results of IEDB categories showing the p-value, and minimal, maximum and median of the p-values in the cross-validation.

			p-value	Cross-validation min p-value	Cross-validation max p-value	Cross-validation median p-value
Allergy						
Food						
	Plants					
		Fabaceae	3E-04	2E-04	0.001	4E-04
Enviroment						
	Plants					
		Amaranthaceae	0.02	0.02	0.06	0.03
		Betulaceae	0.07	0.02	0.39	0.05
		Cupressaceae	0.19	0.03	0.35	0.21
		Timothy Grass	0.85	0.64	0.95	0.82
		Other Grass	0.72	0.55	0.87	0.72
	Fungi					
		Aspergillus	3E-07	1E-07	5E-05	1E-06
		Other Fungi Allergy	0.09	0.04	0.28	0.12
	Animals					
		Insects (V + CR)	0.07	0.01	0.43	0.06
		Arachnid (HDM)	0.68	0.50	0.99	0.62
		Mammals Allergy (cat, dog and others)	2E-12*	1E-13	6E-10	1E-11
Autoimmunity						
		Diabetes	0.94	0.70	0.99	0.94
		RheumatoidArthritis	0.003*	1E-04	0.011	0.004
		Gluten	2E-16*	2E-16	1E-11	0.001
		Multiple Sclerosis	0.001	0.002	0.03	0.005
Infectious Disease						
Virus						
	ssRNA (-) strand virus					
		Influenza AH1N1	1E-09*	1E-11	5E-07	2E-09
		Influenza AH3N2	4E-05*	8E-09	0.003	6E-05
		Other Influenza A	0.008*	0.003	0.03	0.008
		Influenza B	0.31	0.10	0.62	0.32
		Paramyxoviridae	0.27	0.07	0.67	0.27
	ssrNA (+) strand virus					
		Hepatitis C	6E-04*	7E-05	0.02	4E-04
		Dengue	4E-04*	4E-05	0.005	8E-04
		West Nile	0.03*	0.002	0.17	0.03
		Japanese Encephalitis	0.49	0.20	0.74	0.50
		Yellow Fever	0.008	0.002	0.05	0.01
		Coronavirus	0.13	0.05	0.38	0.10
	Retrotranscribing virus					
		Hepatitis B	3E-08*	2E-09	2E-06	3E-07
	dsDNA virus					
		Adenovirus	9E-04	2E-04	0.003	0.002
		Alphaherpesviridae	0.42	0.10	0.75	0.45
		Betaherpesviridae	0.007	0.002	0.09	0.02
		Gammaherpesviridae	0.25	0.010	0.71	0.30
		Poxviridae	0.37	0.11	0.84	0.38
		Papillomavirus	0.02*	0.006	0.07	0.03
Bacteria						
	Actinobacteria/ proteobacteria					
		Alphaproteobacteria	0.02*	0.008	0.11	0.03
		Betaproteobacteria	2E-16*	2E-16	5E-09	2E-16
	Fimmicutes/ other bacteria					
		Clostridiales	0.02*	0.006	0.07	0.02
		Other Bacilli	0.14	0.07	0.38	0.14
Eukaryote						
	Alveolata					
		Plasmodium	1E-08	2E-10	2E-06	2E-08

No asterisk in the p-values denotes epitopes having significant median similarity score lower than the non-epitopes.

* Categories with epitope similarity scores higher than the non-epitopes.

To further validate these observations, we analyzed manually curated sets of dominant epitopes, derived from allergens from Timothy Grass (TG), Ragweed (RAG), house dust mites (HDM), and cockroaches (CR). In general, the epitope sets were selected to encompass the majority of the responses with a limited number of epitopes. We also included manually curated non-epitopes, defined on the basis that these peptides did not induce responses in any of donors tested in the studies in which the positive epitopes were defined. Table 3 presents the results of the analysis of these sets of epitopes (the general matching epitope set from Table 1 is also shown for reference).

**Table 3:** Results of manually curated categories showing the number of epitopes, non-epitopes and associated p-value.

	Epitopes	Non Epitope	p-value
Cupressaceae*	23	752	0.19
Manually curated RAG	30	366	0.02
Timothy Grass*	547	12182	0.85
Manually curated TG	59	889	0.67
Insects (V + CR)*	112	2079	0.07
Manually curated CR	71	1107	6E-13
Arachnid (HDM)*	164	4466	0.68
Manually curated HDM	52	15	0.12

*Results from Table 1 showing results from automatically generated epitope sets of corresponding and matching origin are shown for reference and comparison purpose.

No asterisk in the p-values denotes epitopes having significant median similarity score lower than the non-epitopes.

In most cases the more dominant epitopes had more significant p-values. In the case of the IEDB-derived allergen epitope sets, the Ragweed and Cockroach sets did not achieve significance, whereas the dominant sets did. Therefore, we identified ten categories for which a “tolerogenic” influence was detected.

### Resolution of epitope source and microbiome similarity relationships

The above results indicate that in certain instances microbiome similarity might exert an “inflammatory” influence, while in other instances microbiome similarity might be linked to a “tolerogenic” influence. To investigate this issue further we selected the 14 and 10 categories, for which the significant p-values were noted, to be associated with enhanced “inflammatory” immunogenicity and decreased “tolerogenic” immunogenicity, respectively.

Next, we selected for each category the top 10% highest BLOSUM score epitopes for the inflammatory categories and non-epitopes for the tolerogenic categories. This was done considering highest BLOSUM scores of the epitopes in which the epitopes were more similar than non-epitopes; conversely, in the case where the non-epitope were more similar than the epitopes the highest BLOSUM scores of the non-epitopes was considered. For each of the epitope or non-epitope sequences we selected the hits (homologous sequences in the human microbiome) with a score above the threshold determined by the epitope or non-epitope median of max BLOSUM scores for the respective category. We next counted how frequently each microbiome microorganism was represented in these hits. The hits were then mapped back to the genus of origin from the microbiome sequence sets. Finally, we tabulated for each indication the frequency of hits mapped to each microbiome genus and compared them to the frequency of all 15-mers in the whole microbiome dataset.

An example of this type of analysis for the “Mammal Allergens” category is shown in Table 4. We find that 13 genera were associated with more than 4 epitope-similar hits above the median of the non-epitopes (BLOSUM score > 0.588). For these 13 genera we assessed the significance of differences with the overall microbiome 15-mer frequencies by chi-square probability. As shown in in Table 4, for nine of them significant differences were detected, even after Bonferroni correction.

**Table 4:** Genera of mammalian allergy categories with more than 4 hits.

Genus	Hits	Percentage in Category	Control Percentage	p-value of chi-square	p-value of Bonferroni correction
Streptococcus	49	18.7	6.62	1E-14	1E-13
Parvimonas	5	1.91	0.12	4E-14	5E-13
Finegoldia	5	1.91	0.18	7E-09	9E-08
Bacteroides	5	1.91	12.07	7E-07	9E-06
Enterococcus	56	21.37	11.53	1E-06	1E-05
Oribacterium	7	2.67	0.47	2E-06	3E-05
Fusobacterium	14	5.34	1.85	7E-05	9E-04
Sphingobacterium	5	1.91	0.39	6E-04	7E-03
Eubacterium	7	2.67	0.99	0.02	0.02
Prevotella	5	1.91	3.01	ns	ns
Lactobacillus	13	4.96	3.48	ns	ns
Lachnospiraceae	8	3.05	1.99	ns	ns
Clostridium	15	5.73	4.53	ns	ns

The number of hits per genus and its percentage over the total of 15-mer peptides analyzed (n = 262) per category are indicated. p-values for the chi-square and after Bonferroni correction are also shown.

### Subsets of microbial genus associated with epitope modulation

We next compiled the results of the analysis outlined above for each of the various epitope categories. Table 5 lists the forty-one genera for which a significant Bonferroni-corrected enrichment was detected, in at least 50% of either the epitope or non-epitope modulated categories. In this table, each genus is shown together with the number of instances in which it was found enriched in each of the 14 epitope and 10 non-epitope categories (8 IEDB categories, Cockroach and Ragweed).

**Table 5:** Genera found to be enriched in the inflammatory and tolerogenic categories and overall.

	Epitopes (14 categories)		Non-Epitopes (10 categories)		Overall (24 categories)	
	Enriched	% enriched	Enriched	% enriched	Enriched	% enriched
Fusobacterium	6	42.86	9	90	15	62.5
Streptococcus	6	42.86	9	90	15	62.5
Clostridium	6	42.86	9	90	15	62.5
Bacteroides	8	57.14	6	60	14	58.33
Bifidobacterium	5	35.71	6	60	11	45.83
Propionibacterium	3	21.43	8	80	11	45.83
Staphylococcus	3	21.43	7	70	10	41.67
Enterococcus	3	21.43	7	70	10	41.67
Prevotella	2	14.29	8	80	10	41.67
Neisseria	1	7.14	8	80	9	37.5
Klebsiella	2	14.29	7	70	9	37.5
Escherichia	5	35.71	4	40	9	37.5
Enterobacter	4	28.57	5	50	9	37.5
Corynebacterium	3	21.43	5	50	8	33.33
Lactobacillus	2	14.29	6	60	8	33.33
Coprobacillus	4	28.57	3	30	7	29.17
Lachnospiraceae	1	7.14	6	60	7	29.17
Citrobacter	3	21.43	4	40	7	29.17
Actinomyces	1	7.14	6	60	7	29.17
Acinetobacter	4	28.57	3	30	7	29.17
Finegoldia	2	14.29	5	50	7	29.17
Oribacterium	2	14.29	5	50	7	29.17
Eubacterium	1	7.14	5	50	6	25
Erysipelotrichaceae	0	0	6	60	6	25
Veillonella	1	7.14	5	50	6	25
Parvimonas	0	0	6	60	6	25
Helicobacter	1	7.14	5	50	6	25
Parabacteroides	0	0	6	60	6	25
Mobiluncus	1	7.14	5	50	6	25
Rothia	0	0	6	60	6	25
Providencia	2	14.29	4	40	6	25
Aeromonas	0	0	6	60	6	25
Paenibacillus	1	7.14	5	50	6	25
Dermacoccus	0	0	5	50	5	20.83
Haemophilus	1	7.14	4	40	5	20.83
Afipia	1	7.14	4	40	5	20.83
Atopobium	1	7.14	4	40	5	20.83
Megasphaera	1	7.14	4	40	5	20.83
Proteus	2	14.29	3	30	5	20.83
Myroides	0	0	5	50	5	20.83
Brevibacterium	0	0	5	50	5	20.83
Rhodococcus	0	0	4	40	4	16.67

In instances where the non-epitopes were most similar to the human microbiome (“tolerogenic” categories), the genera *Fusobacterium*, *Streptococcus* and *Clostridium* were enriched in more than 90% of the non-epitopes categories (9/10 or more of the instances). Conversely, the genus *Bacteroides* was associated with the “inflammatory categories” with Bonferroni corrected significant values in at least 60% of the epitope categories (8/14 or more of the instances). The most remarkable finding was, however, that the same genus tended to be enriched in either “inflammatory” or “tolerogenic” categories, suggesting that no clear inflammatory vs. tolerogenic categories can be established at the genus level. Indeed, the genus *Fusobacterium*, *Streptococcus*, *Bacteroides*, *Clostridium*, *Bifidobacterium* and *Propionibacterium* were enriched in hits from both tolerogenic and inflammatory categories. A complete checkerboard analysis showing the genus and each of the 14 epitope and 10 non-epitope categories, respectively, the frequencies in epitopes or non-epitopes, control set, and associated p-values after Bonferroni correction is shown in S9 Table.

### Microbiome peptides homologous to dominant epitopes are associated with constitutive IL-10 production

We next reasoned that the fact that similar microbiome categories might be associated with inflammatory or tolerogenic influence is consistent with microbiome-epitope specific T cells existing in the host in a tolerant and quiescent state. Exposure to the cross-reactive pathogen-derived epitopes might result in an inflammatory or tolerogenic outcome depending on the milieu and the co-stimulation associated with each exposure, and the particular microbiome niche occupied by the microbes that encode the homologous epitopes.

**Fig 3:**
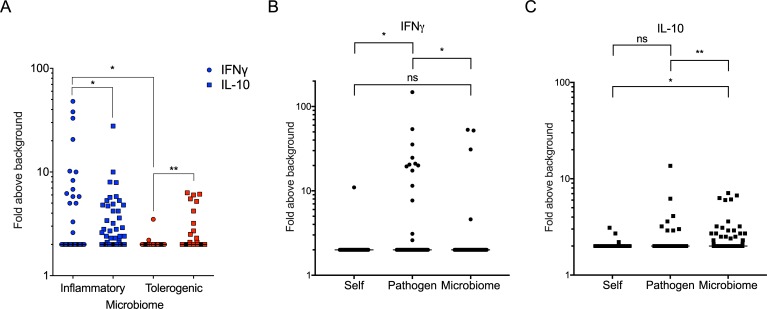
Microbiome peptides homologous to dominant epitopes are associated with constitutive IL-10 production. (A) Combined responses to microbiome derived epitopes for the inflammatory (blue) and tolerogenic (red). (B, C) Combined response to self, pathogen and microbiome derived epitopes for (B) IL-10 and (C) IFNγ. Response is expressed as fold above background. Each dot represents one donor/category combination. IFNγ (dots) and IL-10 (squares). Median ± interquartile range is shown. Two-tailed Mann-Whitney, *, p≤0.05, **, p≤0.01.

To test this hypothesis, high BLOSUM score hits (derived as described in the methods) were next tested for immunogenicity in human PBMC representing a diverse set of HLAs. From the 10 “tolerogenic” (excluding Rheumatoid Arthritis, for which too few homologous peptide hits were available) and 14 “inflammatory” categories, the top ten hits for each category were selected for analysis. Since ten high BLOSUM score peptide hits were selected for each of the 23 categories (Table 6), this corresponded to a total of 230 human microbiome peptides. Pools of peptides corresponding to each category were tested for recognition in an *ex vivo* Fluorospot assay measuring IFNγ and IL-10. The microbiome derived epitopes produced a significant amount of IL-10 for both inflammatory and tolerogenic categories (p = 0.02 and p = 0.001, respectively). Conversely, significant IFNγ production was only detected in the case of the inflammatory, but not the tolerogenic category (p = 0.006) (Fig 3A).

**Table 6:** Tolerogenic versus inflammatory categories.

Tolerogenic categories	Inflammatory categories
Adenovirus	Alphaproteobacteria
Amaranthaceae	Betaproteobacteria
Aspergillus	Clostridales
Betaherpesviridae	Dengue
Cockroach	Gluten
Fabaceae	Hepatitis B
Multiple Sclerosis	Hepatitis C
Plasmodium	Influenza A H1N1
Cupressaceae	Influenza A H3N2
Yellow Fever	Other Influenza A
	Mammals Allergy
	Papillomavirus
	West Nile virus

These data is consistent with microbiome-epitope specific T cells existing in the host in a tolerant state, associated with constitutive IL-10 production. We further hypothesized that this mode of recognition is distinct from the tolerance induced by self-sequences at the level of thymus education (since microbiome peptides are limited or absent in the fetal and newborn stage, and generally not expressed in the thymus). To address this point, we tested three sets of peptides: 1) The microbiome sequences most homologous to dominant epitopes (BLOSUM score >0.6, n = 230), 2) the dominant epitopes themselves (n = 230 as above), and 3) self-sequences most homologous to the microbiome (BLOSUM score >0.7, n = 95) and also predicted to be high affinity promiscuous HLA binders [24]. As shown in Fig 3B and 3C IL-10 production was associated with microbiome peptides homologous to known epitopes, but not the homologous epitopes derived from common pathogens (p = 0.0036). Conversely, IFNγ production was associated with the pathogen epitopes, but not their microbiome homologs (p = 0.03). Finally, self-peptides that were homologous to these microbiome sequences were not associated with either IFNγ or IL-10 production.

### Epitope and non-epitope homologies in microbiota from dust samples from households of cockroach allergic and non-allergic children

We hypothesized that in cockroach allergies, where the microbiome is potentially blunting allergen-specific immune responses, differences at the level of the microbiome associated with allergic versus non-allergic individuals might result in a broader allergen-specific T cell repertoire, increasing the potential for allergen T cell recognition and associated immunopathology. To test this, we analyzed 16S rRNA-based microbiota data derived from dust collected from households of cockroach allergic and non-allergic children [26].

The collection of 16S rRNA sequences derived from allergic and non-allergic microbiomes were analyzed according to Okuda et al. [21]. The phylogeny of each sequence is represented in a taxonomy map and the genome content is predicted by the use of the KEGG orthology (KO) identifiers. Based on each microbiota (allergic and non-allergic) we derived 41 KOs and associated 15-mers unique to allergic microbiota and 150 KOs and associated 15mers unique to non-allergic microbiomes.

**Fig 4:**
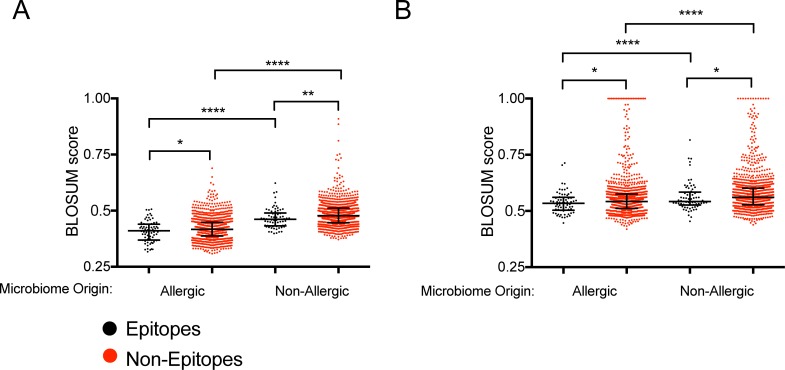
Dust allergen epitopes are less conserved in microbiome derived from allergic and non-allergic households. Maximum BLOSUM score for each epitope/non-epitope per (A) KEGG ontology and (B) proteome in each group. Epitopes (black) and non-epitopes (red) for allergic (left in both graphs) and non-allergic (right in both graphs) households. Two-tailed Mann-Whitney, *, p≤0.05, **, p≤0.01, ****, p≤0.0001.

We next analyzed the set of epitope and non-epitope sequences [13] described above. A BLOSUM score was calculated for each epitope and non-epitope sequence when compared to 15-mers from KOs unique to allergic microbiomes (41) and KOs unique to non-allergic microbiomes (150). The results demonstrate (Fig 4A) that the non-epitopes are more conserved in both allergic and non-allergic KOs (p = 0.04, p = 0.002 respectively), when compared to the epitopes counterpart. Previous analysis [20] demonstrated that the microbiome of non-allergic individuals is significantly more diverse than the microbiome of allergic ones. The four-fold greater number of KOs in the non-allergic group than in the allergic group is reflected in a greater probability of finding matches for any given peptide due to a larger set of sequences. Thus, the tolerogenic effect of the microbiome may be more pronounced in non-allergic individuals, because of the greater diversity of the associated microbiome.

To verify this result with a different approach, taxonomy maps, obtained as described by Okuda et al. [21], for the allergic and non-allergic house dust microbiota were used to generate a list of taxa found in each microbiota. The NCBI taxonomy IDs for each of the species were used to retrieve sequences of proteins for each organism, and derive 36 allergic specific species proteomes, and 61 non-allergic specific species proteomes. The maximum BLOSUM scores were calculated for the epitopes/non-epitopes per each organism proteome. The results, shown in Fig 4B, are consistent with our previous findings. Epitopes were found to be significantly (p = 0.02) less conserved in the proteomes of both organisms unique to allergic dust and non-allergic dust.

## Discussion

Our results show that microbial sequence similarity is a relevant variable to be considered when assessing immunogenicity. Sequence similarity might influence and explain downregulation and tolerogenic immune responses versus "inflammatory" immune reactions to potentially cross-reactive antigens/allergens. The data presented herein provides an extension of the concept of how self/non-self cross-reactivity shapes the repertoire of an individual’s reactivity. First, it provides an extension of the concept of immunological tolerance beyond the sequences encoded by the individual host genome. It is well appreciated that T cells recognizing self-sequences capable of binding self MHC are largely eliminated by central tolerance, and those that do escape the process are actually often associated with regulatory activity, leading to the concept of Tregitopes [27, 28]. Our data suggests the concept of “self” expands beyond the sequences encoded in the genome, to the sequences of the myriad of microorganisms that cohabitate within the living space provided in the human body. This is expected to be the result of peripheral tolerance, where T cells mounting an inflammatory immune responses against non-self antigen can be suppressed by regulatory immune mechanisms intended to limit inflammation in the absence of real danger to the host.

We emphasize that the mechanism for microbiome-based modulation of immune responses described herein is based on sequence homology between specific microbiome sequences and potential epitope sequences from allergens, pathogens or autoantigens. As such, this is different and distinct from other general means by which the microbiome has been described to modulate immune responses. Bacteria present in the microbiome secrete soluble factors and metabolites and these bioactive compounds affect the function of intestinal epithelium and mucosal immune cells, resulting in production of cytokines and related factors [29, 30]. Microbiome antigens and metabolites have been proposed to induce peripherally derived regulatory T cells. TGFβ, retinoic acid, microbial antigens and microbial metabolites promote differentiation and expansion of the peripherally derived Tregs from naive CD4+ T cells [29, 30]. The antigen specificity of these Tregs has not been established, and it is unclear how many of those are microbiome specific, and for which antigens and peptides.

In addition, and in parallel to general immunomodulatory effects, we propose an analogous mechanism based on sequence similarity and molecular cross-reactivity. Indeed, microbiome antigens would be expected, like any other antigen (self or pathogen), to be processed by host APCs which will result in the generation of HLA bound peptides. Furthermore, these peptides are not expected to be presented in the thymus during early ontogeny and would therefore not be subject to central tolerance, and would thus have the potential of being recognized as non-self epitopes. Yet, under normal conditions these microbiome-derived epitopes are not associated with effector type responses, presumably because of peripheral tolerance mechanisms. We propose herein that these mechanisms controlling and regulating potential responses to HLA class II bound and microbiome-derived peptides generated by antigen processing of the microbiome microorganism must necessarily extend to highly homologous epitopes encoded in other protein sequences of pathogen, allergen or autoantigen origin. We propose that the microbiome influences the host immune reactivity both by general immunomodulatory mechanisms and antigen-specific modulation of immune responses, and that both mechanisms act in parallel and in a potentially synergistic fashion. Microbial sequence similarities in subsequent heterologous T cell responses may have profound clinical implications on several levels: (i) heterologous T cell immunity between microbes and self-antigens may extend the concept that (early life) exposure to certain microbes favors the development of clinical immunological tolerance. In addition to unspecific effects promoted by microbial compounds and metabolites [31], here we propose an additional strategy on the level of T cell specificity. (ii) Dependent on the local cytokine milieu this level of cross-reactivity may also result in an inflammatory response. This is an unexpected finding which has also been recently described by Atarashi et al. [32]. This observation suggests that microbiome-specific T cells normally exist in the peripheral T cell repertoire, and depending on the additional milieu and context may exert an inhibitory tolerogenic effect, or on the contrary in particular conditions have an inflammatory effect. Here we have used IFNγ and IL-10 as surrogates for tolerogenic and inflammatory types of immune responses. Whether the inflammatory type is solely dependent on IFNγ remains to be investigated.

In our study, we did not assay for IL-4 or IL-5. However, most of the curated allergen epitope sets from the IEDB (all previously published data), and in particular the cockroach epitopes analyzed in more detail here, have been defined as allergen-specific epitopes on the basis Th2 cytokine production (among them IL-4 and IL-5). Assaying for a broader set of cytokines would be of interest, an in this context, also assaying for IL-17 would be of significant interest.

One additional point to clarify is whether the distribution of HLA class II influence the responses to antigen peptides. The HLA class II alleles are extremely diverse [33]. The sample size of 10 individuals investigated here is therefore too small to draw any conclusions regarding whether certain alleles influence the responses. Future studies would be necessary to directly address this point.

We also examined whether microbiome modulation of antigen specific responses would be HLA class II-specific, or would also extend to HLA class I. Preliminary analysis did not detect significant effect in the case of known class I epitopes (data not shown). This result is in agreement with the hypothesis that the effects detected are based on the internalization and presentation of microbiome derived epitopes through the exogenous HLA class II presentation pathway.

In the present study, no non-inflammatory, non-tolerogenic peptides were tested. These peptides could represent a useful control since they would be expected to be associated with an intermediate response pattern.

The fact that microbiome homology might alter and modulate the reactivity of pathogen or allergen specific T cells was at first surprising. However, several studies have demonstrated that sequence homology can modulate T cell responses, in instances such as epitope sequences conserved across different herpes viruses or allergen genera and several others [4, 34–36]. This also applies for viral antigens. Virus-mediated T cell cross-reactivity has been shown for allo-antigens derived from both related and non-related pathogens [3, 37–41], as well as auto-antigens [42]. We have recently demonstrated T cell cross-reactivity between influenza virus-derived peptides and common allergens such as ovalbumin. Such influenza-specific Th1 T cells prevent the development of a Th2-like immune response against the food allergen at the level of T cell cross-reactivity [5]. Furthermore, we reported a recent example in which the reactivity of *Mycobacterium tuberculosis* specific T cells appears to be regulated by microbiome sequences, and that antibiotic treatment with the capacity to alter microbiome composition alters most profoundly T cell reactivity against those mycobacterial sequences highly homologous to the microbiome [36].

One interesting aspect of the results is that some pathogens were in the “tolerogenic” category. These results were at first unexpected, but it should be noted that the “tolerogenic” label refers to the microbiome homology. In those instances, apparently, the microbiome contains sequences that are highly homologous to the pathogen’s genome, and T cells reactive against those crossreactive epitopes are inactivated potentially by peripheral tolerance. As a result, the epitopes recognized by the host immune system are by default those that do not share as much sequence homology with the microbiome.

In our studies, we relied on a rather stringent definition of sequence homology/similarity, utilizing the BLOSUM matrix [19]. It is likely that this approach might even underestimate the potential impact of sequence homology on microbiome cross-reactive modulation. In fact, several studies reported high levels of cross-reactivity even in instances where little primary sequence homology was demonstrable, including cross-reaction between phylogenetically distant viruses [43], and demonstration of memory T cells reacting against HIV sequences in HIV unexposed individuals [3].

The degree of similarities detected in our studies are certainly influenced by the breadth of metagenomic databases, which have only just started to be developed. Indeed, the Human Microbiome Project database currently represents samples from relatively few, primarily American, individuals and therefore likely underestimates the true diversity of microbial genes. However, our study reports relative differences in degrees of similarity between epitopes and non-epitopes from a given origin. The fact that epitopes are more or less similar to the microbiome than non-epitopes is not likely to be affected by the number of microbiome sequences considered.

The genera *Fusobacterium*, *Streptococcus* and *Clostridium* were enriched in more than 90% of the non-epitope categories from “tolerogenic” categories. Conversely, the genus *Bacteroides* was more frequently associated with “inflammatory categories”. The most remarkable finding was however that the same genera tended to be enriched in both “inflammatory” and “tolerogenic” categories. Indeed, the genus *Fusobacterium*, *Streptococcus*, *Bacteroides*, *Clostridium*, *Bifidobacterium* and *Propionibacterium* were enriched in hits from both tolerogenic and inflammatory categories. These results are compatible with the notion formulated above that microbiome–specific T cells normally found in the peripheral T cell repertoire, depending on the additional milieu and context, might exert either a tolerogenic or inhibitory effect.

In conclusion, microbial epitopes may influence the immune responses to antigens of similar sequences, no matter whether the antigens are derived from pathogens, allergens or hosts. This is a key point of interest of our study, however, more studies are necessary to further elaborate on this conclusion, and in particular to clarify the mechanisms involved. These observations have implications for different disease manifestations in humans. The above-mentioned study in the context of antibiotic treatment in tuberculosis illustrates an example of how microbiome homology can alter the pattern of immune responses. The data presented in this study related to microbiome homology and cockroach allergen T cell epitope repertoire in allergic and non-allergic children suggest that exposure to different microbiome compositions can lead to repertoire differences associated with differential disease susceptibility. Whether this type of observation will be expanded to additional disease settings remains to be established, but it is nevertheless possible that differential repertoire compositions influenced by microbiome homology could be exploited for diagnostic or therapeutic applications.

## Supporting information

S1 Table Categories and their validated HLA class II epitopes from the IEDB. (XLSX)

S2 Table Categories and their control sets for negative epitopes. (XLSX)

S3 Table Sets of dominant epitopes. (XLSX)

S4 Table Sets of negative control epitopes for dominant epitopes. (XLSX)

S5 Table Peptides selected for experimental testing—Tolerogenic. (XLSX)

S6 Table Peptides selected for experimental testing—Inflammatory. (XLSX)

S7 Table Peptides selected for experimental testing–self. (XLSX)

S8 Table HLA type of study subjects. (XLSX)

S9 Table Genus, frequencies and associated p-values for each of the epitope and non-epitope categories. (XLSX)
